# Supplementary material for: Study protocol for investigating the performance of an automated blood test measuring GFAP and UCH-L1 in a prospective observational cohort of patients with mild traumatic brain injury: European BRAINI study
Source: BMJ Open. 2021 Feb 25;11(2):e043635. doi: 10.1136/bmjopen-2020-043635 (PMC7908910; doi:10.1136/bmjopen-2020-043635)
Supplement: Author's manuscript [file bmjopen-2020-043635.draft_revisions.pdf]

BMJ Open is committed to open peer review. As part of this commitment we make the peer review history of every article we publish publicly available.

When an article is published we post the peer reviewers' comments and the authors' responses online. We also post the versions of the paper that were used during peer review. These are the versions that the peer review comments apply to.

The versions of the paper that follow are the versions that were submitted during the peer review process. They are not the versions of record or the final published versions. They should not be cited or distributed as the published version of this manuscript.

BMJ Open is an open access journal and the full, final, typeset and author-corrected version of record of the manuscript is available on our site with no access controls, subscription charges or pay-per-view fees (http://bmjopen.bmj.com).

If you have any questions on BMJ Open's open peer review process please email info.bmjopen@bmj.com

BMJ Open

Predictive performance of an automated assay for GFAP and UCH-L1 blood concentrations in mild Traumatic BRAIN Injury: European BRAINI study

Journal:	*BMJ Open*
Manuscript ID	bmjopen-2020-043635
Article Type:	Protocol
Date Submitted by the Author:	09-Aug-2020
Complete List of Authors:	Richard, Marion; Universite Grenoble Alpes, Department of Anaesthesia and Intensive Care, Centre Hospitalier Universitaire Grenoble Alpes, Grenoble Institut des Neurosciences, INSERM, U1216, 38000<br>Lagares, Alfonso; Hospital Universitario 12 de Octubre Servicio de Neurocirugia, imas12; Universidad Complutense de Madrid, Surgery<br>Bondanese, Victor; bioMerieux SA, Clinical Unit<br>de la Cruz, Javier; Instituto de investigación imas12, Hospital Universitario 12 de Octubre, SAMID<br>Mejan, Odile; bioMerieux SA, Clinical Unit<br>Pavlov, Vladislav; bioMerieux SA, Medical Affairs<br>Payen, Jean-François ; Universite Grenoble Alpes, Department of Anaesthesia and Intensive Care, Centre Hospitalier Universitaire Grenoble Alpes, Grenoble Institut des Neurosciences, INSERM, U1216, 38000
Keywords:	ACCIDENT & EMERGENCY MEDICINE, NEUROSURGERY, TRAUMA MANAGEMENT, Computed tomography < RADIOLOGY & IMAGING

SCHOLARONE™
Manuscripts

I, the Submitting Author has the right to grant and does grant on behalf of all authors of the Work (as defined in the below author licence), an exclusive licence and/or a non-exclusive licence for contributions from authors who are: i) UK Crown employees; ii) where BMJ has agreed a CC-BY licence shall apply, and/or iii) in accordance with the terms applicable for US Federal Government officers or employees acting as part of their official duties; on a worldwide, perpetual, irrevocable, royalty-free basis to BMJ Publishing Group Ltd ("BMJ") its licensees and where the relevant Journal is co-owned by BMJ to the co-owners of the Journal, to publish the Work in this journal and any other BMJ products and to exploit all rights, as set out in our licence.

The Submitting Author accepts and understands that any supply made under these terms is made by BMJ to the Submitting Author unless you are acting as an employee on behalf of your employer or a postgraduate student of an affiliated institution which is paying any applicable article publishing charge ("APC") for Open Access articles. Where the Submitting Author wishes to make the Work available on an Open Access basis (and intends to pay the relevant APC), the terms of reuse of such Open Access shall be governed by a Creative Commons licence – details of these licences and which Creative Commons licence will apply to this Work are set out in our licence referred to above.

Other than as permitted in any relevant BMJ Author's Self Archiving Policies, I confirm this Work has not been accepted for publication elsewhere, is not being considered for publication elsewhere and does not duplicate material already published. I confirm all authors consent to publication of this Work and authorise the granting of this licence.

For peer review only

Predictive performance of an automated assay for GFAP and UCH-L1 blood concentrations in mild Traumatic BRAIN Injury: European BRAINI study

Marion Richard[1], Alfonso Lagares[2], Victor Bondanese[3], Javier De la Cruz[4], Odile Mejan[3], Vladislav Pavlov[5], Jean-François Payen[1], BRAINI Investigators

[1] Univ. Grenoble Alpes, Department of Anaesthesia and Intensive Care, Centre Hospitalier Universitaire Grenoble Alpes, Grenoble Institut des Neurosciences, INSERM, U1216, 38000 Grenoble, France

[2] Servicio de Neurocirugía, Hospital Universitario 12 de Octubre, Universidad Complutense de Madrid, Instituto de Investigación imas12, Madrid, Spain

[3] bioMérieux, Clinical Unit, Chemin de l'Orme, 69280 Marcy l'Etoile, France

[4] Instituto de Investigación imas12, Hospital Universitario 12 de Octubre, SAMID Madrid, Spain

[5] bioMérieux, Medical Affairs, Chemin de l'Orme, 69280 Marcy l'Etoile, France

Corresponding Author:

Prof. Alfonso Lagares, Servicio de Neurocirugía, Hospital Universitario 12 de Octubre, Universidad Complutense de Madrid, Instituto de Investigación imas12, Madrid, Spain. Avda de Cordoba s/n, Madrid 28041. Email: alfonlag@ucm.es

For peer review only

ABSTRACT

Introduction: Mild traumatic brain injury (mTBI) is a common cause of clinical consultation in the Emergency Department. Patients with mTBI may undergo brain CT scans based on clinical criteria. However, the proportion of patients with brain lesions on CT is very low. Two serum biomarkers, glial fibrillary acidic protein (GFAP) and ubiquitin carboxy-terminal hydrolase L1 (UCH-L1), have been shown to discriminate patients regarding the presence or absence of brain lesions on initial CT scan when assessed within the first 12 hours after TBI. However, the current technique for measuring serum concentrations of GFAP and UCH-L1 is manual and time consuming, which may hinder its use in routine clinical practice. This study assesses the diagnostic accuracy of an automated assay for the measurement of serum GFAP and UCH-L1 in a cohort of mTBI patients who received a CT scan as the standard of care.

Methods and analysis: This is a prospective multicentre observational study of 1760 mTBI patients recruited in France and Spain across 12 participating sites. Adult patients with an initial Glasgow Coma Scale score of 13-15 and a brain CT-scan underwent blood sampling within 12 hours after TBI. The primary outcome measure is the diagnostic performance of an automated assay measuring serum concentrations of GFAP and UCH-L1 for discriminating between patients with positive and negative findings on brain CT-scans. Secondary outcome measures include the performance of these two biomarkers in predicting the neurological status and quality of life at 1 week and 3 months after the trauma.

Ethics and dissemination: Ethics approval was obtained by the Institutional Review Board of Sud-Ouest Outre Mer III in France (Re#2019-A01525-52) and Hospital 12 de Octubre in Spain (Re#19/322). The results will be presented at scientific meetings and published in peer-reviewed publications.

Trial registration number (ClinicalTrials.gov): NCT04032509

Key words: Traumatic brain injury, biomarkers, diagnostic techniques and procedures, GFAP, UCH-L1, serum, computerized tomography, prognosis.

For peer review only

STRENGTHS AND LIMITATIONS OF THE STUDY

- This is a large prospective multicentre study to validate the value of serum biomarkers GFAP and UCH-L1 in predicting brain CT-scan findings after mTBI.
- The additional value of these two biomarkers will be assessed regarding neurological outcome and quality of life.
- Variability in the management of mTBI patients and in the indications of CT-scans may influence the rate of positive CT scans in the different participating centres, which may have an impact on the statistical power of the study.

For peer review only

INTRODUCTION

Mild traumatic brain injury (mTBI), as defined by a Glasgow Coma Scale (GCS) score of 13-15 [1], represents a frequent admission in the Emergency Department (ED) [2–4]. The initial management includes a non-contrast brain computed tomography (CT) scan if the patient meets specific conditions. However, the prevalence of CT-detected abnormalities is less than 10% among mTBI patients, and less than 1% of them will require neurosurgical procedures. Efforts have been made for decades to optimize the indications for brain CT scans after mTBI such as the New Orleans Criteria [5] and Canadian Head CT Rule [6] as well as national guidelines such as the French guidelines [7]. However, a certain variability exists among physicians regarding CT scan indications and some situations post-TBI may be confusing, e.g. intoxicated patients or patients with hearing loss or speech disturbances. As a consequence, up to 40% of CT scans prescribed in EDs would actually do not follow guideline recommendations [8], reflecting a substantial CT overuse.

Clinical decision rules for initial CT scan can be optimized with the use of an objective parameter that is easily and rapidly assessed. This could be achieved using blood concentrations of brain-damage biomarkers. Among candidates, serum protein S100B is the only biomarker used in Europe. The Scandinavian guidelines for the initial management of mild and moderate head injuries in adults provide recommendations for the use of S100B to rule out the need for head CT in mTBI. Multicentre validation of Scandinavian Guidelines is currently in progress in Sweden (**ClinicalTrials.gov, 2017, NCT03280485*). Unfortunately, the blood concentration of S100B can be affected by several factors such as multiple trauma, skin colour, presence of melanomas, and its clinical utility is limited by the short half-life (3h) of S100B in blood [9].

Recently, serum levels of ubiquitin carboxy-terminal hydrolase-L1 (UCH-L1) and glial fibrillary acidic protein (GFAP), two brain-specific proteins, were found to be elevated in patients with intracranial lesions visible on CT scans [10,11]. Data from the ALERT-TBI trial showed that serum GFAP and UCH-L1 protein concentrations are able to reliably predict the absence of clinically relevant lesions on CT scan in patients with mTBI [12]. The Banyan Brain Trauma Indicator (*Banyan BTI*™

For peer review only

Package Insert) has obtained FDA-clearance in February 2018. This is a manual immunoassay that measures GFAP and UCH-L1 serum concentrations with a sensitivity (95% lower confidence limit) and negative predictive value (95% lower confidence limit) of 97.5% (93.7%) and 99.6% (98.8%), respectively[13]. However, the manual ELISA technique takes 4 hours to provide results, which is too long for mTBI triage in the ED setting. In addition, the cut-offs used in the ALERT-TBI trial need to be externally validated [12,14].

A faster *in vitro* diagnostic (IVD) technique is then required for a possible use in clinical practice, together with an external validation of the diagnostic accuracy of GFAP and UCH-L1 in mTBI patients [15–18]. An automated assay assessing serum concentrations of GFAP and UCH-L1 (VIDAS® BTI assay, bioMérieux, Marcy l'Etoile, France) has been developed. The primary objective of the study is to evaluate the diagnostic performance of the VIDAS® BTI assay in a prospective multicentre cohort of mTBI patients with respect to their brain CT scan findings. The secondary objectives are to assess the ability of the two biomarkers to predict the neurological status and quality of life at 1 week and 3 months after mTBI.

METHODS AND ANALYSIS

Study design

BRAINI is a prospective, multicentre, observational study in France and Spain.

Study setting

BRAINI includes 11 sites in France within university hospitals (Grenoble, Lyon Edouard-Herriot, Lyon-Sud, Tours, Nantes, Dijon, Poitiers, Montpellier and Toulouse) and non-university hospitals (Annecy and Villefranche-sur-Saone) and 3 sites in Madrid, Spain, including University Hospital 12 de Octubre, University Hospital Gregorio Marañón and Hospital del Tajo. Each centre was chosen based on documentation with regard to patient availability and experience in mild TBI patient management.

Study population

For peer review only

Patients will be included if they meet the following criteria: age >18 years (France) and > 15 years old (Spain), admitted for a mild TBI with GCS score 13-15, requiring brain CT scan as part of standard of care according to the French guidelines [7] or to the in-charge physician in Spain, and 10-mL blood sample obtained as part of routine blood samples within 12 hours after injury.

Patients will be excluded if they have at least one of the following criteria: GCS score 3-12 on admission; time of injury unknown; time since injury exceeding 12 hours; primary admission for non-traumatic neurological disorder (e.g., stroke, spontaneous intracranial hematoma); penetrating head injury; mechanical ventilation; neuropsychiatric and neurologic co-morbidities that might interfere with the assessment of outcomes at 1 week and 3 months; venepuncture not feasible; no brain CT scan; subject under judiciary control; pregnant or breastfeeding woman; or participation in another therapeutic study.

Study outcomes

The primary outcome measure is the performance of the VIDAS BTI assay in terms of sensitivity, specificity, positive predictive value (PPV), negative predictive value (NPV) and their corresponding lower limit of the 95% confidence interval with respect to brain CT scan findings, i.e., positive vs. negative (see below).

Secondary outcomes are measured at 1 week and 3 months post-TBI and include neurological status, i.e., stable or degraded condition, and quality of life assessed using the QOLIBRI questionnaire (quality of life after TBI) [19–21] at 1 week. At 3 months post-TBI, patients will be assessed according to the Extended Glasgow Outcome Scale (GOSE) [22,23], the 5-level EQ-5D version [24], the QOLIBRI scale, and the Rivermead Post-concussion Symptoms Questionnaire (RPQ) [25]. The study design and flow is shown in Fig.1.

Data collection and data monitoring

At each participating site, data will be collected and entered into the web-based electronic CRF (eCRF) (Medsharing, Fontenay-sous-Bois, France) by clinical research associates under the supervision of the site principal investigators. The study database will be created from the eCRF. Trained research coordinators will monitor the data collection. The study will collect demographic and baseline

For peer review only - http://bmjopen.bmj.com/site/about/guidelines.xhtml

information at admission, the reason to prescribe brain CT scan and immediate CT findings by the local radiologist, biological data if indicated by the in-charge physician, a 10-ml study-specific blood sample within 12 hours after trauma, and functional outcomes at 1 week and 3 months. At one week post-TBI, clinical research associates will transmit information regarding the neurological status of the patients to the coordinating centres (University Hospital Grenoble Alpes and University Hospital 12 de Octubre, Madrid). Questionnaires at 3 months will be conducted by trained central outcome assessors during a structured telephone interview. Anonymized data from brain CT images will be transferred to a centralized platform to be evaluated by a trained neuroradiologist. Capture of all data, particularly the functional outcome assessments and CT scan analysis, will be conducted by persons who are blinded to the results of the two biomarkers.

Data analysis: CT scan

Brain CT scans are performed as part of the patient standard of care and uploaded to a secure web central database (SHANOIR-INRIA) for archive and central analysis. CT scan findings are classified as CT-negative or CT-positive by the local and one central reading. In case of disagreement between them, an additional central CT-reading will be performed by a third radiologist for final adjudication. The criteria for CT positivity are crucial because the primary objective is related to the CT results (positive/negative). Criteria for CT-positive findings are as follows: a CT scan classified as type II or greater according to the Traumatic Coma Databank (TCDB) classification (range I-V), or a CT scan with the presence of the following lesions: a) epidural haematoma, b) subdural haematoma, c) subarachnoid haemorrhage, d) intraventricular haemorrhage, e) contusion, f) petechial haemorrhage or g) any finding related to diffuse axonal injury (DAI) and depressed skull fracture. Linear skull fractures will be recorded but not used for the definition of CT positive lesions.

Data analysis: biomarkers

The 10-ml blood sample will be centrifuged without delay and frozen at -80°C. Serum samples will be transferred to the central storage facility (bioMérieux R&D BioBank, Marcy l'Étoile, France). All measurement procedures will be conducted independently

For peer review only

based on the clinical and CT-status of the patients. The samples will be batch-analysed using fully automated instruments (VIDAS® 3, bioMérieux) with kits to detect the serum concentrations of UCH-L1 and GFAP.

Statistical considerations

We formulate the hypothesis that the automated assay measuring the serum concentrations of GFAP and UCH-L1 will have a sensitivity of at least 97% and an NPV of at least 99% to rule out the need for CT scan with accuracy. Assuming a prevalence of 11% of positive brain CT scans, the enrolment of 176 patients with positive CT-scans and 1424 patients with negative CT-scans is required to achieve these indices of performance. . Assuming a 10% rate of loss to follow-up, the study recruitment target is set to 1760 patients in France and in Spain.

For the primary objective, the previously published cut-off values for GFAP and UCH-L1 will be externally validated with respect to the CT scan findings. The levels of GFAP and UCH-L1 obtained with the VIDAS BTI will also be analysed according to the neurological outcome, the presence of posttraumatic symptoms and quality of life (secondary objective). To test whether these biomarkers are independent prognostic factors, multivariable analysis will be conducted using logistic regression for dichotomized values and linear regression for quantitative values. In addition, prediction models will be developed to assess the contribution of biomarkers to existing diagnostic and prognostic tools.

ETHICS AND DISSEMINATION

Ethics approval was obtained from the CEIm (Comité Ético de Investigación Clínica) of Hospital 12 de Octubre on 20 July 2019, Madrid (Re#19/322) and the study started in August 2019. In France, the protocol was approved by the Institutional Review Board of Sud-Ouest Outre Mer III on 14 November 2019 (Re#2019-A01525-52). The study began in France in Grenoble on 29 November 2019. The National Commission on Informatics and Liberty (France) gave its approval on 31 January 2020 (Re#919443).

For peer review only

The results of this study will be presented at national and international meetings and published in peer-reviewed journals. Patients will not be individually notified regarding the results of the study. The principal publication from the study will be in the name of the BRAINI investigators with full credit assigned to all active, collaborating investigators, research coordinators and institutions.

DISCUSSION

The protocol and future results of the current study should be analysed taking into account previously published data. In the large CENTER-TBI cohort study, the addition of serum GFAP, measured within 24 h following mTBI was found to improve the prediction of CT abnormalities [18]. Although the combination of GFAP and UCH-L1 did not enhance this performance, compared to GFAP alone [18], it should be noted that GFAP and UCH-L1 were measured within 24-h after TBI using a research-use only (RUO) assay with poor agreement between replicates of biomarker assessments. The present study will evaluate the diagnostic accuracy of the combination of GFAP and UCH-L1, measured within 12 hours post-TBI, using an automated Vidas IVD-platform. In addition, an external validation of the cut-off values for these two biomarkers will be performed. Finally, GFAP and UCH-L1 will be assessed regarding their possible prediction of mid-term neurological outcome and quality of life.

There are some limitations with the BRAINI protocol. First, variability in mTBI management and CT ordering may be expected in Spain, as there is no consensus regarding clinical decision rules for ordering CT, and inter-centre differences may exist in France as well. This may in turn influence the CT-positive prevalence across the sites. Second, differences might occur between local and central CT readings. To mitigate this risk, an additional independent central CT reading will be performed for final adjudication. Third, only mTBI patients with brain CT scans will be included in this study. Patients discharged from the ED following clinical examination without CT scan will not be included; therefore, their GFAP and UCH-L1 concentrations will not be captured. As a consequence, the value of GFAP and UCH-L1 in all TBI presentations will not be determined.

For peer review only

REFERENCES

1 Teasdale G, Jennett B. ASSESSMENT OF COMA AND IMPAIRED CONSCIOUSNESS: A Practical Scale. *Lancet* 1974;**304**:81–4. doi:10.1016/S0140-6736(74)91639-0

2 Levin HS, Diaz-Arrastia RR. Diagnosis, prognosis, and clinical management of mild traumatic brain injury. Lancet Neurol. 2015;**14**:506–17. doi:10.1016/S1474-4422(15)00002-2

3 Gomez PA, Lobato RD, Lagares A, *et al.* Mild head injury in adults. A review. *Neurocirugia* 2000;**11**.

4 Gómez PA, Lobato RD, Ortega JM, *et al.* Mild head injury: Differences in prognosis among patients with a Glasgow Coma Scale score of 13 to 15 and analysis of factors associated with abnormal CT findings. *Br J Neurosurg* 1996;**10**:453–60. doi:10.1080/02688699647078

5 Haydel MJ, Preston CA, Mills TJ, *et al.* Indications for Computed Tomography in Patients with Minor Head Injury. *N Engl J Med* 2000;**343**:100–5. doi:10.1056/NEJM200007133430204

6 Stiell IG, Wells GA, Vandemheen K, *et al.* The Canadian CT Head Rule for patients with minor head injury. *Lancet* 2001;**357**:1391–6. doi:10.1016/S0140-6736(00)04561-X

7 Jehlé E, Honnart D, Grasleguen C, *et al.* Traumatisme crânien léger (score de Glasgow de 13 à 15): Triage, évaluation, examens complémentaires et prise en charge précoce chez le nouveau-né, l'enfant et l'adulte. *Ann Fr Med d'Urgence* 2012;**2**:199–214. doi:10.1007/s13341-012-0202-4

8 Melnick ER, Szlezak C, Bentley S, *et al.* CT Overuse for Mild Traumatic Brain Injury. *Jt Comm J Qual Patient Saf* 2012;**38**:483–9.

9 Undén J, Ingebrigtsen T, Romner B. Scandinavian guidelines for initial management of minimal, mild and moderate head injuries in adults: An evidence and consensus-based update. *BMC Med* 2013;**11**:50. doi:10.1186/1741-7015-11-50

10 Papa L, Brophy GM, Welch RD, *et al.* Time course and diagnostic accuracy of

For peer review only

glial and neuronal blood biomarkers GFAP and UCH-L1 in a large cohort of trauma patients with and without mild traumatic brain injury. *JAMA Neurol* 2016;**73**:551–60. doi:10.1001/jamaneurol.2016.0039

11 Diaz-Arrastia R, Wang KKW, Papa L, *et al.* Acute biomarkers of traumatic brain injury: Relationship between plasma levels of ubiquitin C-terminal hydrolase-l1 and glial fibrillary acidic protein. *J Neurotrauma* 2014;**31**:19–25. doi:10.1089/neu.2013.3040

12 Bazarian JJ, Biberthaler P, Welch RD, *et al.* Serum GFAP and UCH-L1 for prediction of absence of intracranial injuries on head CT (ALERT-TBI): a multicentre observational study. *Lancet Neurol* 2018;**17**:782–9. doi:10.1016/S1474-4422(18)30231-X

13 Banyan BTI Package Insert. http://banyanbio.com/assets/files/BanyanBTI_IFU_700027_v1.pdf (accessed 1 Jun 2020).

14 Maas AIR, Lingsma HF. ALERT-TBI study on biomarkers for TBI: has science suffered? *Lancet Neurol* 2018;**17**:737–8. doi:10.1016/S1474-4422(18)30275-8

15 Gill J, Latour L, Diaz-Arrastia R, *et al.* Glial fibrillary acidic protein elevations relate to neuroimaging abnormalities after mild TBI. *Neurology* 2018;**91**:E1385–9. doi:10.1212/WNL.0000000000006321

16 Shahjouei S, Sadeghi-Naini M, Yang Z, *et al.* The diagnostic values of UCH-L1 in traumatic brain injury: A meta-analysis. Brain Inj. 2018;**32**:1–17. doi:10.1080/02699052.2017.1382717

17 Yue JK, Yuh EL, Korley FK, *et al.* Association between plasma GFAP concentrations and MRI abnormalities in patients with CT-negative traumatic brain injury in the TRACK-TBI cohort: a prospective multicentre study. *Lancet Neurol* 2019;**18**:953–61. doi:10.1016/S1474-4422(19)30282-0

18 Czeiter E, Amrein K, Gravesteijn BY, *et al.* Blood biomarkers on admission in acute traumatic brain injury: Relations to severity, CT findings and care path in the CENTER-TBI study. *EBioMedicine* 2020;**56**. doi:10.1016/j.ebiom.2020.102785

For peer review only

19 von Steinbüchel N, Wilson L, Gibbons H, *et al.* Quality of Life after Brain Injury (QOLIBRI): scale validity and correlates of quality of life. *J Neurotrauma* 2010;**27**:1157–65. doi:10.1089/neu.2009.1077

20 Castaño-León AM, Navarro-Main B, Gomez PA, *et al.* Quality of Life After Brain Injury: Psychometric Properties of the Spanish Translation of the QoLIBRI. *Eval Heal Prof* 2018;**41**. doi:10.1177/0163278717702696

21 Jourdan C, Bayen E, Pradat-Diehl P, *et al.* A comprehensive picture of 4-year outcome of severe brain injuries. Results from the PariS-TBI study. *Ann Phys Rehabil Med* 2016;**59**:100–6. doi:10.1016/j.rehab.2015.10.009

22 Wilson JTL, Pettigrew LEL, Teasdale GM. Structured interviews for the glasgow outcome scale and the extended glasgow outcome scale: Guidelines for their use. J. Neurotrauma. 1998;**15**:573–80. doi:10.1089/neu.1998.15.573

23 Teasdale GM, Pettigrew LEL, Wilson JTL, *et al.* Analyzing outcome of treatment of severe head injury: A review and update on advancing the use of the Glasgow outcome scale. J. Neurotrauma. 1998;**15**:587–97. doi:10.1089/neu.1998.15.587

24 Herdman M, Gudex C, Lloyd A, *et al.* Development and preliminary testing of the new five-level version of EQ-5D (EQ-5D-5L). *Qual Life Res* 2011;**20**:1727–36. doi:10.1007/s11136-011-9903-x

25 King NS, Crawford S, Wenden FJ, *et al.* The Rivermead Post Concussion Symptoms Questionnaire: a measure of symptoms commonly experienced after head injury and its reliability. *J Neurol* 1995;**242**:587–92. doi:10.1007/BF00868811

For peer review only

Author's contribution: MR, AL, VB, JDC, OM, VP and JFP initiated the study design, wrote the study protocol and drafted the manuscript. All are part of the steering committee of the study. All authors attest to have significantly contributed to refinement of the study protocol and approved the final manuscript. All members of BRAINI contributed to the design and application of the protocol.

The following are BRAINI investigators: Maxime Maigmane[6], Laurent Jacquin[7], Marion Douplat[8], Said Laribi[9], Philippe Pes[10], Patrick Ray[11], Jérémy Guenezan[12], Mustapha Sebbane[13], Frédéric Balen[14], Guillaume Durand[15], Cordelia Abric[16], Podaru Mihai[17],Julian Morales[18], Ana Castuera[18], Mariana Garcia Ponce[19], Maite Cuesta[19]

[6] Emergency Department, University Hospital, Grenoble, France; [7] Hospices Civils of Lyon, Emergency department, Edouard Herriot Hospital, Lyon, France ; [8] Hospices Civils de Lyon, Emergency Department, Lyon, France; [9] Department of Emergency Medicine, Tours University Hospital, Tours, France; [10] Department of Emergency Medicine, University Hospital of Nantes, Nantes, France; [11] Emergency Department, University Hospital of Dijon Bourgogne, Dijon CEDEX, France; [12] CHU de Poitiers, Service des Urgences et SAMU 86, Poitiers, France, Université de Poitiers, Faculté de Médecine et de Pharmacie de Poitiers, Poitiers, France; [13] Department of Emergency Medicine and Prehospital Care, Montpellier University Hospital, Montpellier, France; [14] Emergency Department, Toulouse University Hospital, Toulouse, France; [15] Hôpital Nord-Ouest Villefranche sur Saône, France; [16] CH Annecy Genevois, France; [17] Servicio de Urgencias, Hospital Universitario del Tajo, Madrid, España; [18] Servicio de Urgencias, Hospital Universitario Gregorio Marañón, Madrid, España; [19] Servicio de Neurocirugía, Instituto de Investigación imas 12, Hospital Universitario 12 de Octubre, Madrid, España.

Funding: This study is supported by a grant from the European Institute of Innovation and Technology (EIT) Health (BP 2019-2020). EIT Health is supported by EIT, a body of the European Union.

Competing interests: VB, OM and VP are employees of bioMérieux.

Study status: Recruitment started in August 2019. Study completion is planned in June 2021.

For peer review only

Figure Legend

Fig. 1. Study design and flow of the BRAINI study.

For peer review only

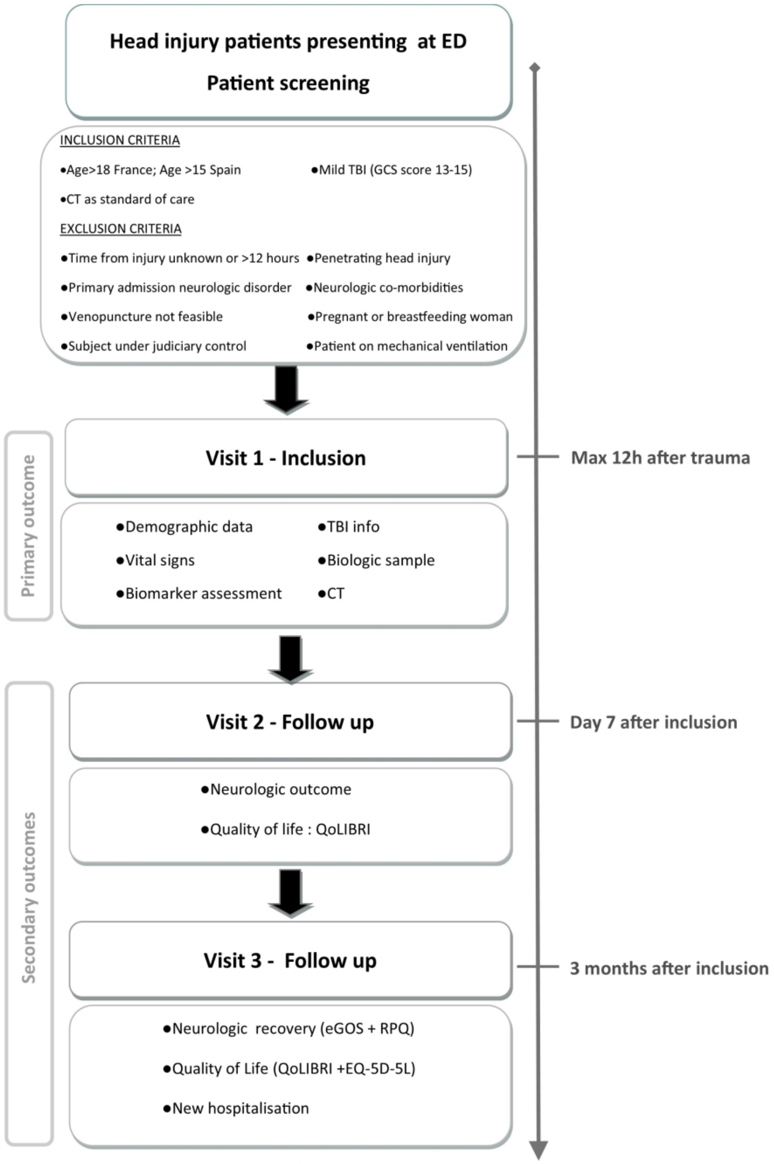

Figure 1. Study design and flow of the BRAINI study.

656x927mm (96 x 96 DPI)

BMJ Open

A study protocol for investigating the performance of an automated blood test measuring GFAP and UCH-L1 in a prospective observational cohort of patients with mild Traumatic BRAIN Injury: European BRAINI study

Journal:	*BMJ Open*
Manuscript ID	bmjopen-2020-043635.R1
Article Type:	Protocol
Date Submitted by the Author:	02-Nov-2020
Complete List of Authors:	Richard, Marion; Universite Grenoble Alpes, Department of Anaesthesia and Intensive Care, Centre Hospitalier Universitaire Grenoble Alpes, Grenoble Institut des Neurosciences, INSERM, U1216, 38000<br>Lagares, Alfonso; Hospital Universitario 12 de Octubre Servicio de Neurocirugia, imas12; Universidad Complutense de Madrid, Surgery<br>Bondanese, Victor; bioMerieux SA, Clinical Unit<br>de la Cruz, Javier; Instituto de investigación imas12, Hospital Universitario 12 de Octubre, SAMID<br>Mejan, Odile; bioMerieux SA, Clinical Unit<br>Pavlov, Vladislav; bioMerieux SA, Medical Affairs<br>Payen, Jean-François ; Universite Grenoble Alpes, Department of Anaesthesia and Intensive Care, Centre Hospitalier Universitaire Grenoble Alpes, Grenoble Institut des Neurosciences, INSERM, U1216, 38000
**Primary Subject Heading**:	Emergency medicine
Secondary Subject Heading:	Emergency medicine, Diagnostics, Neurology
Keywords:	ACCIDENT & EMERGENCY MEDICINE, NEUROSURGERY, TRAUMA MANAGEMENT, Computed tomography < RADIOLOGY & IMAGING

SCHOLARONE™ Manuscripts

I, the Submitting Author has the right to grant and does grant on behalf of all authors of the Work (as defined in the below author licence), an exclusive licence and/or a non-exclusive licence for contributions from authors who are: i) UK Crown employees; ii) where BMJ has agreed a CC-BY licence shall apply, and/or iii) in accordance with the terms applicable for US Federal Government officers or employees acting as part of their official duties; on a worldwide, perpetual, irrevocable, royalty-free basis to BMJ Publishing Group Ltd ("BMJ") its licensees and where the relevant Journal is co-owned by BMJ to the co-owners of the Journal, to publish the Work in this journal and any other BMJ products and to exploit all rights, as set out in our licence.

The Submitting Author accepts and understands that any supply made under these terms is made by BMJ to the Submitting Author unless you are acting as an employee on behalf of your employer or a postgraduate student of an affiliated institution which is paying any applicable article publishing charge ("APC") for Open Access articles. Where the Submitting Author wishes to make the Work available on an Open Access basis (and intends to pay the relevant APC), the terms of reuse of such Open Access shall be governed by a Creative Commons licence – details of these licences and which Creative Commons licence will apply to this Work are set out in our licence referred to above.

Other than as permitted in any relevant BMJ Author's Self Archiving Policies, I confirm this Work has not been accepted for publication elsewhere, is not being considered for publication elsewhere and does not duplicate material already published. I confirm all authors consent to publication of this Work and authorise the granting of this licence.

For peer review only

A study protocol for investigating the performance of an automated blood test measuring GFAP and UCH-L1 in a prospective observational cohort of patients with mild Traumatic BRAIN Injury: European BRAINI study

Marion Richard[1], Alfonso Lagares[2], Victor Bondanese[3], Javier De la Cruz[4], Odile Mejan[3], Vladislav Pavlov[5], Jean-François Payen[1], BRAINI Investigators

[1] Univ. Grenoble Alpes, Department of Anaesthesia and Intensive Care, Centre Hospitalier Universitaire Grenoble Alpes, Grenoble Institut des Neurosciences, INSERM, U1216, 38000 Grenoble, France

[2] Servicio de Neurocirugía, Hospital Universitario 12 de Octubre, Universidad Complutense de Madrid, Instituto de Investigación imas12, Madrid, Spain

[3] bioMérieux, Clinical Unit, Chemin de l'Orme, 69280 Marcy l'Etoile, France

[4] Instituto de Investigación imas12, Hospital Universitario 12 de Octubre, SAMID Madrid, Spain

[5] bioMérieux, Medical Affairs, Chemin de l'Orme, 69280 Marcy l'Etoile, France

Corresponding Author:

Prof. Alfonso Lagares, Servicio de Neurocirugía, Hospital Universitario 12 de Octubre, Universidad Complutense de Madrid, Instituto de Investigación imas12, Madrid, Spain. Avda de Cordoba s/n, Madrid 28041. Email: alfonlag@ucm.es

For peer review only

ABSTRACT

Introduction: Mild traumatic brain injury (mTBI) is a common cause of clinical consultation in the Emergency Department. Patients with mTBI may undergo brain CT scans based on clinical criteria. However, the proportion of patients with brain lesions on CT is very low. Two serum biomarkers, glial fibrillary acidic protein (GFAP) and ubiquitin carboxy-terminal hydrolase L1 (UCH-L1), have been shown to discriminate patients regarding the presence or absence of brain lesions on initial CT scan when assessed within the first 12 hours after TBI. However, the current technique for measuring serum concentrations of GFAP and UCH-L1 is manual and time consuming, which may hinder its use in routine clinical practice. This study assesses the diagnostic accuracy of an automated assay for the measurement of serum GFAP and UCH-L1 in a cohort of mTBI patients who received a CT scan as the standard of care.

Methods and analysis: This is a prospective multicentre observational study of 1760 mTBI patients recruited in France and Spain across 16 participating sites. Adult patients with an initial Glasgow Coma Scale score of 13-15 and a brain CT-scan underwent blood sampling within 12 hours after TBI. The primary outcome measure is the diagnostic performance of an automated assay measuring serum concentrations of GFAP and UCH-L1 for discriminating between patients with positive and negative findings on brain CT-scans. Secondary outcome measures include the performance of these two biomarkers in predicting the neurological status and quality of life at 1 week and 3 months after the trauma.

Ethics and dissemination: Ethics approval was obtained by the Institutional Review Board of Sud-Ouest Outre Mer III in France (Re#2019-A01525-52) and Hospital 12 de Octubre in Spain (Re#19/322). The results will be presented at scientific meetings and published in peer-reviewed publications.

Trial registration number (ClinicalTrials.gov): NCT04032509

Key words: Traumatic brain injury, biomarkers, diagnostic techniques and procedures, GFAP, UCH-L1, serum, computerized tomography, prognosis.

For peer review only

STRENGTHS AND LIMITATIONS OF THE STUDY

- This is a large prospective multicentre study to validate the value of serum biomarkers GFAP and UCH-L1 in predicting brain CT-scan findings after mTBI.
- The additional value of these two biomarkers will be assessed regarding neurological outcome and quality of life.
- Variability in the management of mTBI patients and in the indications of CT-scans may influence the rate of positive CT scans in the different participating centres, which may have an impact on the statistical power of the study.

For peer review only

INTRODUCTION

Mild traumatic brain injury (mTBI), as defined by a Glasgow Coma Scale (GCS) score of 13-15 [1], represents a frequent admission in the Emergency Department (ED) [2–4]. The initial management includes a non-contrast brain computed tomography (CT) scan if the patient meets specific conditions. However, the prevalence of CT-detected abnormalities is less than 10% among mTBI patients, and less than 1% of them will require neurosurgical procedures. Efforts have been made for decades to optimize the indications for brain CT scans after mTBI such as the New Orleans Criteria [5] and Canadian Head CT Rule [6] as well as national guidelines such as the French guidelines [7]. However, a certain variability exists among physicians regarding CT scan indications and some situations post-TBI may be confusing, e.g. intoxicated patients or patients with hearing loss or speech disturbances. As a consequence, up to 40% of CT scans prescribed in EDs would actually do not follow guideline recommendations [8], reflecting a substantial CT overuse.

Clinical decision rules for initial CT scan can be optimized with the use of an objective parameter that is easily and rapidly assessed. This could be achieved using blood concentrations of brain-damage biomarkers. Among candidates, serum protein S100B is the only biomarker used in Europe. The Scandinavian guidelines for the initial management of mild and moderate head injuries in adults provide recommendations for the use of S100B to rule out the need for head CT in mTBI. Multicentre validation of Scandinavian Guidelines is currently in progress in Sweden (**ClinicalTrials.gov, 2017, NCT03280485*). Unfortunately, the blood concentration of S100B can be affected by several factors such as multiple trauma, skin colour, presence of melanomas, and its clinical utility is limited by the short half-life (3h) of S100B in blood [9].

Recently, serum levels of ubiquitin carboxy-terminal hydrolase-L1 (UCH-L1) and glial fibrillary acidic protein (GFAP), two brain-specific proteins, were found to be elevated in patients with intracranial lesions visible on CT scans [10,11]. Data from the ALERT-TBI trial showed that serum GFAP and UCH-L1 protein concentrations are able to reliably predict the absence of clinically relevant lesions on CT scan in patients with mTBI [12]. The Banyan Brain Trauma Indicator (*Banyan BTI™ Package Insert)* has obtained FDA-clearance in February 2018. This is a manual immunoassay that measures GFAP and UCH-L1 serum concentrations with a

sensitivity (95% lower confidence limit) and negative predictive value (95% lower confidence limit) of 97.5% (93.7%) and 99.6% (98.8%), respectively[13]. However, the manual ELISA technique takes 4 hours to provide results, which is too long for mTBI triage in the ED setting. In addition, the cut-offs used in the ALERT-TBI trial need to be externally validated [12,14].

A faster *in vitro* diagnostic (IVD) technique is then required for a possible use in clinical practice, together with an external validation of the diagnostic accuracy of GFAP and UCH-L1 in mTBI patients [15–18]. Automated assays assessing serum concentrations of GFAP and UCH-L1 have been developed on the VIDAS® platform (bioMérieux, Marcy l'Etoile, France). The primary objective of the study is to evaluate the diagnostic performance of the VIDAS® GFAP and VIDAS® UCHL-1 assays in a prospective multicentre cohort of mTBI patients with respect to their brain CT scan findings. The secondary objectives are to assess the ability of the two biomarkers to predict the neurological status and quality of life at 1 week and 3 months after mTBI.

METHODS AND ANALYSIS

Study design

BRAINI is a prospective, multicentre, observational study in France and Spain.

Study setting

BRAINI includes 12 sites in France within university hospitals (Grenoble, Lyon Edouard-Herriot, Lyon-Sud, Tours, Nantes, Dijon, Poitiers, Montpellier, Toulouse and Bordeaux) and non-university hospitals (Annecy and Villefranche-sur-Saone) and 4 sites in Madrid, Spain, including University Hospital 12 de Octubre, University Hospital Gregorio Marañón, Hospital del Tajo and Hospital de La Princesa. Each centre was chosen based on documentation with regard to patient availability and experience in mild TBI patient management.

Study population

Patients will be included if they meet the following criteria: age >18 years (France) and > 15 years old (Spain), admitted for a mild TBI with GCS score 13-15, requiring

For peer review only

brain CT scan as part of standard of care according to the French guidelines [7] or to the in-charge physician in Spain, and 10-mL blood sample obtained as part of routine blood samples within 12 hours after injury.

Patients will be excluded if they have at least one of the following criteria: GCS score 3-12 on admission; time of injury unknown; time since injury exceeding 12 hours; primary admission for non-traumatic neurological disorder (e.g., stroke, spontaneous intracranial hematoma); penetrating head injury; mechanical ventilation; neuropsychiatric and neurologic co-morbidities that might interfere with the assessment of outcomes at 1 week and 3 months; venepuncture not feasible; no brain CT scan; subject under judiciary control; pregnant or breastfeeding woman; or participation in another therapeutic study.

Patients will be included after verification of the eligibility criteria. In France patient non-opposition to participate in the study must be documented. In Spain written informed consent will be obtained, before inclusion in the study, from the patient or next of kin if the patient is not in condition of giving consent.

Study outcomes

The primary outcome measure is the performance of the VIDAS® GFAP and VIDAS® UCHL-1 assays in terms of sensitivity, specificity, positive predictive value (PPV), negative predictive value (NPV) and their corresponding lower limit of the 95% confidence interval with respect to brain CT scan findings, i.e., positive vs. negative (see below).

Secondary outcomes are measured at 1 week and 3 months post-TBI and include neurological status, i.e., stable or degraded condition, and quality of life assessed using the QOLIBRI questionnaire (quality of life after TBI) [19–21] at 1 week. At 3 months post-TBI, patients will be assessed according to the Extended Glasgow Outcome Scale (GOSE) [22,23], the 5-level EQ-5D version [24], the QOLIBRI scale, and the Rivermead Post-concussion Symptoms Questionnaire (RPQ) [25]. The study design and flow is shown in Fig.1.

Data collection and data monitoring

At each participating site, data will be collected and entered into the web-based electronic CRF (eCRF) (Medsharing, Fontenay-sous-Bois, France) by clinical

For peer review only

research associates under the supervision of the site principal investigators. The study database will be created from the eCRF. Trained research coordinators will monitor the data collection. The study will collect demographic and baseline information at admission, the reason to prescribe brain CT scan and immediate CT findings by the local radiologist, biological data if indicated by the in-charge physician, a 10-ml study-specific blood sample within 12 hours after trauma, and functional outcomes at 1 week and 3 months. At one week post-TBI, clinical research associates will transmit information regarding the neurological status of the patients to the coordinating centres (University Hospital Grenoble Alpes and University Hospital 12 de Octubre, Madrid). Questionnaires at 3 months will be conducted by trained central outcome assessors during a structured telephone interview. Anonymized data from brain CT images will be transferred to a centralized platform to be evaluated by a trained neuroradiologist. Capture of all data, particularly the functional outcome assessments and CT scan analysis, will be conducted by persons who are blinded to the results of the two biomarkers.

Data analysis: CT scan

Brain CT scans are performed as part of the patient standard of care and uploaded to a secure web central database (SHANOIR-INRIA) for archive and central analysis. CT scan findings are classified as CT-negative or CT-positive by the local and one central reading. In case of disagreement between them, an additional central CT-reading will be performed by a third radiologist for final adjudication. The criteria for CT positivity are crucial because the primary objective is related to the CT results (positive/negative). Criteria for CT-positive findings are as follows: a CT scan classified as type II or greater according to the Traumatic Coma Databank (TCDB) classification (range I-V), or a CT scan with the presence of the following lesions: a) epidural haematoma, b) subdural haematoma, c) subarachnoid haemorrhage, d) intraventricular haemorrhage, e) contusion, f) petechial haemorrhage or g) any finding related to diffuse axonal injury (DAI) and depressed skull fracture. Linear skull fractures will be recorded but not used for the definition of CT positive lesions.

For peer review only

Data analysis: biomarkers

The 10-ml blood sample for determination of biomarkers will be collected using gel-separator tubes and allowed to clot for 30 minutes at room temperature (18-25ºC) and then centrifuged at 2000G for 15 minutes. Serum will be aliquoted to 1ml cryovials, frozen and stored at -80ºC within two hours of the extraction until shipment on dry ice to the central storage facility (bioMérieux R&D bioBank, Marcy l'Étoile, France). All measurement procedures will be conducted independently based on the clinical and CT-status of the patients. The samples will be batch-analysed using fully automated instruments (VIDAS®, bioMérieux) with kits to detect the serum concentrations of UCH-L1 and GFAP.

bioMérieux will perform analytical performance according to Clinical & Laboratory Standards Institute (CLSI) guidelines including precision study, i.e. repeatability and reproducibility of the assays. In the absence of reference standard and reference measurement procedure for GFAP and UCH-L1, the VIDAS® assays will be traceable to an internal standard. The metrological traceability chain defined in accordance with the standard ISO 17511 will ensure the GFAP and UCH-L1 values assigned to calibrators, product calibrator and patient samples.

Statistical considerations

The Banyan BTI™ test for which Bayan biomarkers got FDA clearance is the only IVD reference assay for GFAP and UCHL-1 so far. It will be used as the reference for the VIDAS® GFAP and VIDAS® UCHL-1 assays.

We formulate the hypothesis that the automated assay measuring the serum concentrations of GFAP and UCH-L1 will have a sensitivity of at least 97% and an NPV of at least 99% to rule out the need for CT scan with accuracy. Assuming a prevalence of 11% of positive brain CT scans, the enrolment of 176 patients with positive CT-scans and 1424 patients with negative CT-scans is required to achieve these indices of performance. . Assuming a 10% rate of loss to follow-up, the study recruitment target is set to 1760 patients in France and in Spain.

For peer review only

Cut-off values for UCH-L1 (327 pg/ml) and GFAP (22 pg/ml) applied in the ALERT-TBI study were fixed for the Banyan BTI™ [12]. We will verify the predictive performances of UCH-L1 and GFAP measured on the VIDAS® instrument with these predefined cut-off values. An analysis on whether alternative cut-offs could provide better performance with the VIDAS® BTI, will also be performed based on BRAINI study data. A method comparison between Banyan BTI™ and VIDAS® BTI will be performed and will contribute to optimal cut-offs selection.

The distribution of biomarker levels will be analysed by age, gender and other predictors. As a secondary objective, the levels of GFAP and UCH-L1 obtained with the VIDAS® assays will also be analysed according to the neurological outcome, the presence of posttraumatic symptoms and quality of life (secondary objective). To test whether these biomarkers are independent prognostic factors, multivariable analysis will be conducted using logistic regression for dichotomized variables and linear regression for quantitative variables. In addition, prediction models will be developed to assess the contribution of biomarkers to existing diagnostic and prognostic tools.

Patient and public involvement

No patient involved.

ETHICS AND DISSEMINATION

Ethics approval was obtained from the CEIm (Comité Ético de Investigación Clínica) of Hospital 12 de Octubre on 20 July 2019, Madrid (Re#19/322) and the study started in August 2019. In France, the protocol was approved by the Institutional Review Board of Sud-Ouest Outre Mer III on 14 November 2019 (Re#2019-A01525-52). The study began in France in Grenoble on 29 November 2019. The National Commission on Informatics and Liberty (France) gave its approval on 31 January 2020 (Re#919443).

The results of this study will be presented at national and international meetings and published in peer-reviewed journals. Patients will not be individually notified regarding the results of the study. The principal publication from the study will be in

For peer review only

the name of the BRAINI investigators with full credit assigned to all active, collaborating investigators, research coordinators and institutions.

DISCUSSION

The protocol and future results of the current study should be analysed taking into account previously published data. In the large CENTER-TBI cohort study, performance of a panel of six blood biomarkers including UCH-L1 and GFAP showed trends in biomarker ability to improve diagnosis, triage and clinical care in TBI in a wide range of contexts of care (Emergency Room, ward admission and ICU) and severities. GFAP, measured within 24 h following mTBI was found to improve the prediction of CT abnormalities [18]. Although the combination of GFAP and UCH-L1 did not enhance this performance, compared to GFAP alone [18], it should be noted that GFAP and UCH-L1 were measured within 24-h after TBI using a research-use only (RUO) assay with poor agreement between replicates of biomarker assessments. No cut-off values for GFAP or UCH-L1 were obtained or predefined in the analysis. The present study will evaluate the diagnostic accuracy on the presence of CT findings of the combination of GFAP and UCH-L1, measured within 12 hours post-TBI, using an automated VIDAS® IVD-platform, in mild TBI patients. In addition, an external validation of the cut-off values for these two biomarkers will be performed. Finally, GFAP and UCH-L1 will be assessed regarding their possible prediction of mid-term neurological outcome and quality of life.

There are some limitations with the BRAINI protocol. First, variability in mTBI management and CT ordering may be expected in Spain, as there is no consensus regarding which clinical decision rules should be used for ordering CT, and inter-centre differences may exist in France as well. This may in turn influence the CT-positive prevalence across the sites. To understand the variability in CT ordering between and within countries and centres, the reason for performing cranial CT will be recorded and analysed in relation to the degree of compliance with clinical decision rules for CT ordering and the percentage of positive CTs in each centre. This information could help understand the generalizability of the results. Second, differences might occur between local and central CT readings. To mitigate this risk, an additional independent central CT reading will be performed for final adjudication.

For peer review only

Third, only mTBI patients with brain CT scans will be included in this study. Patients discharged from the ED following clinical examination without CT scan will not be included; therefore, their GFAP and UCH-L1 concentrations will not be captured. As a consequence, the value of GFAP and UCH-L1 in all TBI presentations will not be determined.

REFERENCES

1 Teasdale G, Jennett B. ASSESSMENT OF COMA AND IMPAIRED CONSCIOUSNESS: A Practical Scale. *Lancet* 1974;**304**:81–4. doi:10.1016/S0140-6736(74)91639-0

2 Levin HS, Diaz-Arrastia RR. Diagnosis, prognosis, and clinical management of mild traumatic brain injury. Lancet Neurol. 2015;**14**:506–17. doi:10.1016/S1474-4422(15)00002-2

3 Gomez PA, Lobato RD, Lagares A, *et al.* Mild head injury in adults. A review. *Neurocirugia* 2000;**11**.

4 Gómez PA, Lobato RD, Ortega JM, *et al.* Mild head injury: Differences in prognosis among patients with a Glasgow Coma Scale score of 13 to 15 and analysis of factors associated with abnormal CT findings. *Br J Neurosurg* 1996;**10**:453–60. doi:10.1080/02688699647078

5 Haydel MJ, Preston CA, Mills TJ, *et al.* Indications for Computed Tomography in Patients with Minor Head Injury. *N Engl J Med* 2000;**343**:100–5. doi:10.1056/NEJM200007133430204

6 Stiell IG, Wells GA, Vandemheen K, *et al.* The Canadian CT Head Rule for patients with minor head injury. *Lancet* 2001;**357**:1391–6. doi:10.1016/S0140-6736(00)04561-X

7 Jehlé E, Honnart D, Grasleguen C, *et al.* Traumatisme crânien léger (score de Glasgow de 13 à 15): Triage, évaluation, examens complémentaires et prise en charge précoce chez le nouveau-né, l’enfant et l’adulte. *Ann Fr Med d’Urgence* 2012;**2**:199–214. doi:10.1007/s13341-012-0202-4

8 Melnick ER, Szlezak C, Bentley S, *et al.* CT Overuse for Mild Traumatic Brain

For peer review only

Injury. *Jt Comm J Qual Patient Saf* 2012;**38**:483–9.

9 Undén J, Ingebrigtsen T, Romner B. Scandinavian guidelines for initial management of minimal, mild and moderate head injuries in adults: An evidence and consensus-based update. *BMC Med* 2013;**11**:50. doi:10.1186/1741-7015-11-50

10 Papa L, Brophy GM, Welch RD, *et al.* Time course and diagnostic accuracy of glial and neuronal blood biomarkers GFAP and UCH-L1 in a large cohort of trauma patients with and without mild traumatic brain injury. *JAMA Neurol* 2016;**73**:551–60. doi:10.1001/jamaneurol.2016.0039

11 Diaz-Arrastia R, Wang KKW, Papa L, *et al.* Acute biomarkers of traumatic brain injury: Relationship between plasma levels of ubiquitin C-terminal hydrolase-l1 and glial fibrillary acidic protein. *J Neurotrauma* 2014;**31**:19–25. doi:10.1089/neu.2013.3040

12 Bazarian JJ, Biberthaler P, Welch RD, *et al.* Serum GFAP and UCH-L1 for prediction of absence of intracranial injuries on head CT (ALERT-TBI): a multicentre observational study. *Lancet Neurol* 2018;**17**:782–9. doi:10.1016/S1474-4422(18)30231-X

13 Banyan BTI Package Insert. http://banyanbio.com/assets/files/BanyanBTI_IFU_700027_v1.pdf (accessed 1 Jun 2020).

14 Maas AIR, Lingsma HF. ALERT-TBI study on biomarkers for TBI: has science suffered? *Lancet Neurol* 2018;**17**:737–8. doi:10.1016/S1474-4422(18)30275-8

15 Gill J, Latour L, Diaz-Arrastia R, *et al.* Glial fibrillary acidic protein elevations relate to neuroimaging abnormalities after mild TBI. *Neurology* 2018;**91**:E1385–9. doi:10.1212/WNL.0000000000006321

16 Shahjouei S, Sadeghi-Naini M, Yang Z, *et al.* The diagnostic values of UCH-L1 in traumatic brain injury: A meta-analysis. Brain Inj. 2018;**32**:1–17. doi:10.1080/02699052.2017.1382717

17 Yue JK, Yuh EL, Korley FK, *et al.* Association between plasma GFAP concentrations and MRI abnormalities in patients with CT-negative traumatic

For peer review only

brain injury in the TRACK-TBI cohort: a prospective multicentre study. *Lancet Neurol* 2019;**18**:953–61. doi:10.1016/S1474-4422(19)30282-0

18 Czeiter E, Amrein K, Gravesteijn BY, *et al.* Blood biomarkers on admission in acute traumatic brain injury: Relations to severity, CT findings and care path in the CENTER-TBI study. *EBioMedicine* 2020;**56**. doi:10.1016/j.ebiom.2020.102785

19 von Steinbüchel N, Wilson L, Gibbons H, *et al.* Quality of Life after Brain Injury (QOLIBRI): scale validity and correlates of quality of life. *J Neurotrauma* 2010;**27**:1157–65. doi:10.1089/neu.2009.1077

20 Castaño-León AM, Navarro-Main B, Gomez PA, *et al.* Quality of Life After Brain Injury: Psychometric Properties of the Spanish Translation of the QoLIBRI. *Eval Heal Prof* 2018;**41**. doi:10.1177/0163278717702696

21 Jourdan C, Bayen E, Pradat-Diehl P, *et al.* A comprehensive picture of 4-year outcome of severe brain injuries. Results from the PariS-TBI study. *Ann Phys Rehabil Med* 2016;**59**:100–6. doi:10.1016/j.rehab.2015.10.009

22 Wilson JTL, Pettigrew LEL, Teasdale GM. Structured interviews for the glasgow outcome scale and the extended glasgow outcome scale: Guidelines for their use. J. Neurotrauma. 1998;**15**:573–80. doi:10.1089/neu.1998.15.573

23 Teasdale GM, Pettigrew LEL, Wilson JTL, *et al.* Analyzing outcome of treatment of severe head injury: A review and update on advancing the use of the Glasgow outcome scale. J. Neurotrauma. 1998;**15**:587–97. doi:10.1089/neu.1998.15.587

24 Herdman M, Gudex C, Lloyd A, *et al.* Development and preliminary testing of the new five-level version of EQ-5D (EQ-5D-5L). *Qual Life Res* 2011;**20**:1727–36. doi:10.1007/s11136-011-9903-x

25 King NS, Crawford S, Wenden FJ, *et al.* The Rivermead Post Concussion Symptoms Questionnaire: a measure of symptoms commonly experienced after head injury and its reliability. *J Neurol* 1995;**242**:587–92. doi:10.1007/BF00868811

For peer review only

Author's contribution: MR, AL, VB, JDC, OM, VP and JFP initiated the study design, wrote the study protocol and drafted the manuscript. All are part of the steering committee of the study. All authors attest to have significantly contributed to refinement of the study protocol and approved the final manuscript. All members of BRAINI contributed to the design and application of the protocol.

The following are BRAINI investigators: Maxime Maignan[6], Laurent Jacquin[7], Marion Douplat[8], Said Laribi[9], Philippe Pes[10], Patrick Ray[11], Jérémy Guenezan[12], Mustapha Sebbane[13], Frédéric Balen[14], Guillaume Durand[15], Cordelia Abric[16], Cédric Gil-Jardiné[17], Podaru Mihai[18],Julian Morales[19], Ana Castuera[19], Mariana Garcia Ponce[20], Maite Cuesta[20], Jose A. F. Alén[21]

[6] Emergency Department, University Hospital, Grenoble, France; [7] Hospices Civils of Lyon, Emergency department, Edouard Herriot Hospital, Lyon, France ; [8] Hospices Civils de Lyon, Emergency Department, Lyon, France; [9] Department of Emergency Medicine, Tours University Hospital, Tours, France; [10] Department of Emergency Medicine, University Hospital of Nantes, Nantes, France; [11] Emergency Department, University Hospital of Dijon Bourgogne, Dijon, France; [12] CHU de Poitiers, Service des Urgences et SAMU 86, Poitiers, France, Université de Poitiers, Faculté de Médecine et de Pharmacie de Poitiers, Poitiers, France; [13] Department of Emergency Medicine and Prehospital Care, Montpellier University Hospital, Montpellier, France; [14] Emergency Department, Toulouse University Hospital, Toulouse, France; [15] Hôpital Nord-Ouest Villefranche sur Saône, France; [16] CH Annecy Genevois, France; [17] Emergency Department, University Hospital Bordeaux, France ; [18] Servicio de Urgencias, Hospital Universitario del Tajo, Madrid, Spain; [19] Servicio de Urgencias, Hospital Universitario Gregorio Marañón, Madrid, Spain; [20] Servicio de Neurocirugía, Instituto de Investigación imas 12, Hospital Universitario 12 de Octubre, Madrid, Spain; [21] Servicio de Neurocirugia, Hospital Universitario de la Princesa, Madrid, Spain.

Funding: This study is supported by a grant from the European Institute of Innovation and Technology (EIT) Health (BP 2019-2020). EIT Health is supported by EIT, a body of the European Union.

bioMérieux is responsible for the development and manufacturing of the VIDAS® GFAP and VIDAS® UCHL-1 assays. bioMérieux will provide in-kind support to the

For peer review only

study by supplying the assays for the measurement of UCH-L1 and GFAP necessary for this study.

Competing interests: VB, OM and VP are employees of bioMérieux.

Study status: Recruitment started in August 2019. Study completion is planned in June 2021.

For peer review only

Figure Legend

Fig. 1. Study design and flow of the BRAINI study.

For peer review only

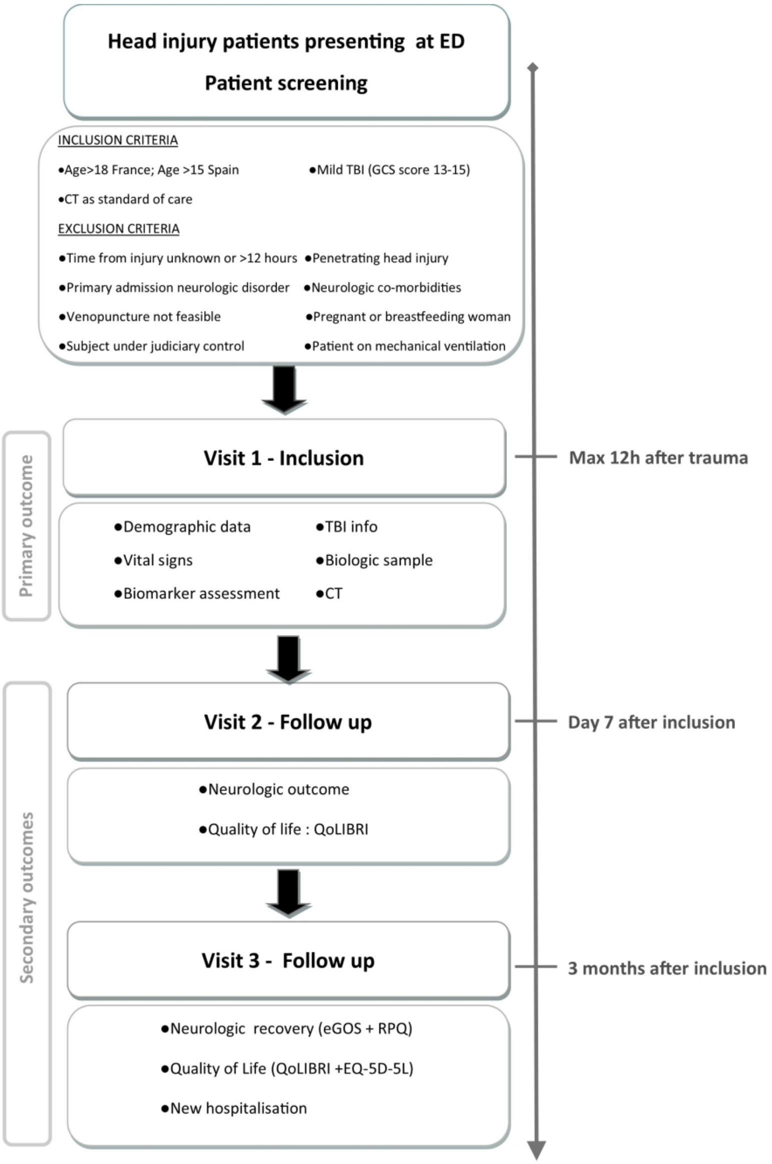

Figure 1. Study design and flow of the BRAINI study.

656x927mm (96 x 96 DPI)
